# Post-Synapse Model Cell for Synaptic Glutamate Receptor (GluR)-Based Biosensing: Strategy and Engineering to Maximize Ligand-Gated Ion-Flux Achieving High Signal-to-Noise Ratio

**DOI:** 10.3390/s120101035

**Published:** 2012-01-18

**Authors:** Akito Tateishi, Sarah K. Coleman, Satoshi Migita, Kari Keinänen, Tetsuya Haruyama

**Affiliations:** 1 Department of Biological Functions and Engineering, Kyushu Institute of Technology, Kitakyushu Science and Research Park, Fukuoka, 808-0196, Japan; E-Mails: tateishi-akito@edu.life.kyutech.ac.jp (A.T.); migita.met@tmd.ac.jp (S.M.); 2 Department of Biosciences, Division of Biochemistry and Biotechnology, Viikki Biocenter, University of Helsinki, Helsinki, Finland; E-Mails: sarah.coleman@helsinki.fi (S.K.C.); kari.keinanen@helsinki.fi (K.K.)

**Keywords:** cell-based biosensors, high through-put analysis (HTA), organ function model, post-synapse model cell, cell engineering, ionotropic glutamate receptor, signal-to-noise ratio

## Abstract

Cell-based biosensing is a “smart” way to obtain efficacy-information on the effect of applied chemical on cellular biological cascade. We have proposed an engineered post-synapse model cell-based biosensors to investigate the effects of chemicals on ionotropic glutamate receptor (GluR), which is a focus of attention as a molecular target for clinical neural drug discovery. The engineered model cell has several advantages over native cells, including improved ease of handling and better reproducibility in the application of cell-based biosensors. However, in general, cell-based biosensors often have low signal-to-noise (S/N) ratios due to the low level of cellular responses. In order to obtain a higher S/N ratio in model cells, we have attempted to design a tactic model cell with elevated cellular response. We have revealed that the increase GluR expression level is not directly connected to the amplification of cellular responses because the saturation of surface expression of GluR, leading to a limit on the total ion influx. Furthermore, coexpression of GluR with a voltage-gated potassium channel increased Ca^2+^ ion influx beyond levels obtained with saturating amounts of GluR alone. The construction of model cells based on strategy of amplifying ion flux per individual receptors can be used to perform smart cell-based biosensing with an improved S/N ratio.

## 1. Introduction

Cultured mammalian cell-based biosensors have been developed to determine the effects of extracellular stimuli on cellular function in basic research fields, and drug discovery [1,2]. These are a smart way to screen lead compounds on the basis of their effects on metabolic/catabolic cascades [3–5]. However, in general, cell-based biosensors have low signal-to-noise (S/N) ratio due to the low signal of cellular responses. To solve this problem, not only must the sensitivity of sensors be improved but also the tactic biosensor cells need to be engineered using e.g., genetic engineering [6,7], cellular engineering [8,9], and cell culture engineering [5,10].

A conjugative technology which combined with chemical sensors and organ function model has been proposed [4]. On the basis of this concept, we demonstrated a post-synaptic function model that consists of genetically engineered cells designed to minimize the fluctuation of data in cell-based assay for neural drug discovery [11]. The post-synapse model cells express ionotropic glutamate receptor (GluR) on their membrane surface. As such, these cells can recognize either the agonistic and antagonistic activity on the basis of a change in the ion flux profile [12]. In addition, our post-synapse model cells grow faster and are easier to handle than cultured neuronal cells under experimental conditions [11]. Therefore, these model cells have several advantages as a functional model for cell-based biosensors for high through-put analysis (HTA).

Recently, many researchers have attempted to develop cell-based biosensors using receptor-expressing cells because receptors are one of the main targets for neural drug discovery [13,14]. However, owing to the low level of cellular signals, receptor-expressing cell-based biosensors often have low S/N ratio. In order to improve the S/N ratio in receptor-expressing cell-based biosensors, it is important to amplify the cellular responses.

In the case of ionotropic receptor-expressing cells, the cellular response, *i.e.*, ion flux on the whole cell, was controlled by the number of receptors on the cell membrane and the ion flux level per individual receptor. Therefore, in order to amplify the cellular responses on receptor-expressing cells, one can increase either the receptor expression level or the ion flux level per individual receptors. In this study, we investigate and demonstrate a new design for post-synapse model cells realizing a high S/N ratio. The approach involves attempting to increase the ion flux level per individual receptor, which is directly linked to amplification of cellular responses (ion influx through GluR).

## 2. Experimental Section

### 2.1. Chemicals

l-Glutamic acid and 2,3-dihydroxy-6-nitro-7-sulfamoyl-benzo[*f*]quinoxaline-2,3-dione (NBQX), were purchased from Wako Chemical (Tokyo, Japan). Margatoxin was purchased from Peptide Institute, Inc (Osaka, Japan). The acetoxymethyl form of Fura-2 was purchased from Invitrogen (Carlsbad, CA, USA). All chemicals of guaranteed experimental grade.

### 2.2. Cell Culture and Transfection

COS7 cells were cultured in Dulbecco’s modified Eagles medium (DMEM) containing 10% FBS, 100 U/mL penicillin, and 100 μg/mL streptomycin. Flag-tagged GluA4 (Δ22-402) [15], a miniaturized version of GluA4 receptor lacking the N-terminal domain, was constructed in pIRES2-EGFP vector (Clontech Laboratories, Mountain View, CA, USA) as described previously [11]. Rat voltage-gated Shaker potassium channel, Kv1.3 [16] was amplified by polymerase chain reaction. The amplicon was ligated to the *XhoI* and *EcoRI* sites using pIRES2-DsRed plasmid vector (Clontech). The expression plasmids were transfected into COS7 cells using Xfect™ (Invitrogen). After transfection, the cells were cultured in DMEM with 10% FBS, 100 U/mL penicillin, 100 μg/mL streptomycin. NBQX (100 μM) was added in order to suppress the potential toxicity of glutamate present in the medium.

### 2.3. Immuno-Fluorescence Staining

The engineered cells were cultured on a fibronectin coated coverslips. The cells were fixed with 3% paraformaldehyde and used for immuno-staining (nonpermeabilized condition, surface staining). In the case of permeabilized condition, the cells were treated with 0.05% Triton-X solution in PBS after paraformaldehyde fixation. Nonspecific binding was blocked by incubation in SuperBlock blocking buffer (Pierce Biotechnology, Rockford, IL, USA). The cells were labeled with monoclonal rabbit anti-FLAG IgG (Sigma, St. Louis, MO, USA; 5 μg/mL) followed by rhodamine-conjugated anti-rabbit IgG secondary antibody (Sigma; 7 μg/mL). They were then examined using an epifluorescence microscope (Nikon, Japan). Pictures were collected using an air-cooled CCD camera and analyzed using the AQUA COSMOS software (Hamamatsu Photonics, Japan).

### 2.4. Fluorescence Measurement of Ion Flux

GluA4 channels are permeable to both sodium and calcium influxes by its agonistic function [17]. We monitored calcium influx using calcium ion indicator dye, Fura-2. The dye for post-synapse model cells was prepared and loaded as described previously [11]. The synapse model cells were loaded with Fura-2 AM in Hank’s buffered salt solution, and incubated for 1 h at 37 °C. Intracellular calcium ion imaging was performed every 8 s using an epifluorescence microscope with air-cooled CCD camera and analyzed using AQUA COSMOM software. Fluorescence (emission at 510 nm) was recorded at 8 s intervals for excitation at 340 and 380 nm, and the fluorescence ratio (R = I_340nm_/I_380nm_). The data were compared using Student’s *t* test.

## 3. Results and Discussion

**Figure 1.**
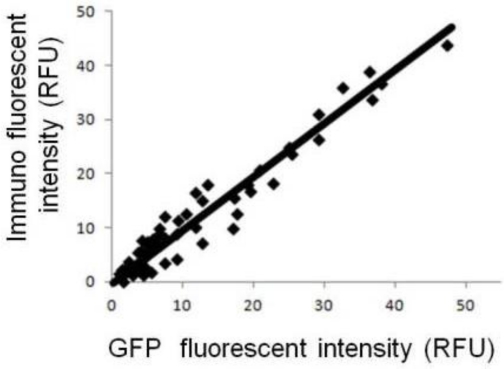
Relationship between fluorescent intensity of GFP and total GluR immunofluorescence.

This research is intended to promote and demonstrate a strategic engineering approach for amplification of ligand-gated ion influx on post-synapse model cells. Initially, we investigated what happens when GluR expression is simply increased. COS7 cells were transfected with expression plasmid encoding GluR and IRES-linked GFP marker. Owing to the function of the IRES sequence, GluR expression was correlated with the cellular fluorescence intensity derived from GFP. Figure 1 shows the relationship between fluorescent intensity of GFP and immuno-staining under the cellular membrane-permeabilized conditions. Under these conditions, the antibody could bind to both intracellular GluR and GluR on the cell membrane surface, and the total fluorescent intensity reflects the GluR expression level. As the GFP fluorescence intensity correlates with the fluorescence intensity of immuno-staining, GFP can be used as a reporter of the level of GluR expression.

**Figure 2.**
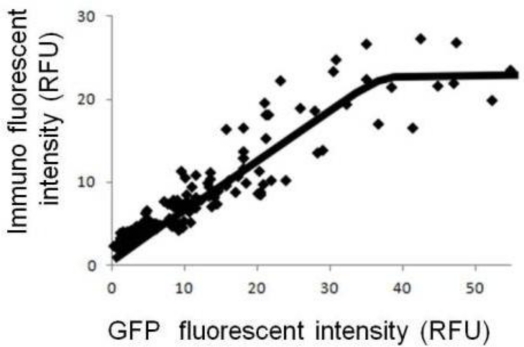
Relationship between fluorescent intensity of GFP (coexpressed with GluR) and immunofluorescence staining of GluR on cell surface (without cellular membrane-permeabilization).

Figure 2 shows the relationship between the fluorescent intensity of GFP and the immuno-staining without cellular membrane-permeabilization. Under such conditions, fluorescence intensity from immuno-staining indicates the number of GluR molecules on the cell membrane. The data indicate that saturation occurred: the number of GluR on the cell membrane increased as GluR expression level increased up to a certain level, but then leveled off. In our previous study, we demonstrated that cellular responses on the model cells were saturated at a certain level of GluR expression [11]. These data suggest that the number of GluR on the cell membrane has reached the maximal value obtainable under the experimental conditions. In the case of higher GluR-expressing cells, GluR is evenly displayed on cell surface and variation in cellular response can be reduced. Therefore, the reproducibility of cell-based sensors will be improved by using higher GluR-expressing cells. However, limitation of the number of GluR on the cell surface leads to a limitation on the maximal amplification of ion flux. Therefore, increasing the GluR expression level is not necessarily directly connected to amplification of the cellular responses.

**Figure 3.**
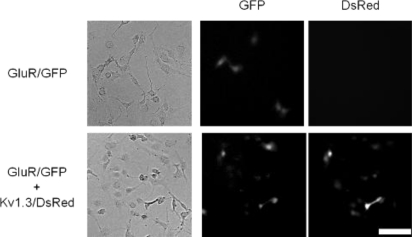
Visualization of the post-synapse model cell expressing GluR and Kv1.3, visualized by GFP and DsRed markers also present in the IRES vectors for the respective proteins. The scale bar represents 100 μm.

Next, we tried to verify the usefulness of an alternative strategy: in increasing the ion flux per individual receptor. Maintenance of normal membrane potential should provide driving force for sustained ion flux, and therefore, we coexpressed a voltage-gated potassium channel potassium channel subunit, Kv1.3 [16] with GluR [18]. Kv1.3 should thus keep the membrane potential below reversal potential and facilitate enhanced ion flux through GluR. In the coexpressing cells, the expression level of GluR was confirmed by the fluorescence of GFP and expression of Kv1.3 was confirmed by the fluorescence of DsRed (Figure 3).

**Figure 4.**
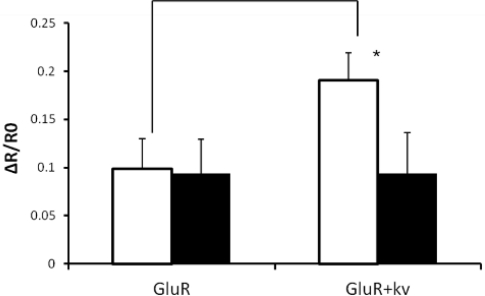
Ion flux level of post-synapse model cells that express only GluR or coexpress GluR and Kv1.3 (2 mM glutamate application [open]: 2 mM glutamate application after 10 nM margatoxin treatment [filled]). Ca^2+^ influx was monitored by changes in fura-2 340/380 nm ratio (R) normalized to baseline (Δ R/R0). The data represent the mean ± SD with (*) indicating *P* < 0.01.

The Ca^2+^ influx of GluR-expressing cells or GluR-Kv1.3-cotransfected cells was analyzed using Fura-2 (Figure 4). Glutamate-triggered ion flux level increased when Kv1.3 was also present. This increase of ion flux was prevented by Kv1.3 blocker, margatoxin [19,20], suggesting that activation of Kv1.3 contributes to the increased ion flux through GluR. Without Kv1.3 activation, membrane potential is depolarized by the ion influx through GluR, leading to a reduction in the driving force of ion influx.

## 4. Conclusions

The strategic design and construction of post-synapse model cells for cellular biosensing, and potentially useful in drug screening, is described. In this study, we focused on obtaining a high S/N ratio for Ca^2+^ influx through GluR. A simple increase of the expression level of receptors was found to be insufficient. However, coexpression with Kv1.3, as demonstrated here, allowed membrane potential to be kept below the reversal potential and resulted in enhanced ion flux responses through GluR.

In conclusion, we demonstrate a strategy to increase the ion flux per individual receptor and improve the S/N ratio in ligand-gated ion channel-expressing cell-based biosensors. We also demonstrated that the reproducibility of cell-based sensors will be improved by using higher GluR-expressing cell. These approaches may provide a smart way to build post-synapse model cell-based biosensors for HTA drug discovery.
